# Improving the Corrosion Resistance of Zn-Rich Epoxy Coating with Three-Dimensional Porous Graphene

**DOI:** 10.3390/polym15214302

**Published:** 2023-11-01

**Authors:** Zhihong Qin, Yinqiang Su, Yang Bai, Hangqi Lu, Tao Peng, Huifeng Zhong, Tao Chen, Xusheng Du

**Affiliations:** 1 The Fifth Engineering Co., Ltd., MBEC, Jiujiang 332001, China; qinzhihong1987@163.com; 2 Zhuhai Communication Group, Zhuhai 519000, China; laosi188@163.com (Y.S.); lhq345000233@163.com (H.L.); pengtao13709690425@163.com (T.P.); zhfmerry@163.com (H.Z.); ct18666940946@163.com (T.C.); 3 CCCC-SHB Fifth Engineering Co., Ltd, Xi’an 710119, China; baiyang20051987@163.com; 4 Institute of Advanced Wear & Corrosion Resistant and Functional Materials, Jinan University, Guangzhou 510632, China

**Keywords:** graphene sheets, graphene foam, zinc-rich epoxy coating, corrosion resistance, cathodic protection

## Abstract

To improve the corrosion inhibition of zinc-rich epoxy (ZRE) composite coatings and shed light on the influence of the spatial structure of graphene fillers on the coatings’ performance, three-dimensional graphene (3DG) and a conventional graphene sheet (G) were used to modify the ZRE composite paint, respectively. The effect of introducing the 2D G fillers on the anti-corrosion behavior of ZRE was studied comprehensively, and its optimal content was determined to be 0.5 wt%. Interestingly, it was found that, comparing with 2D graphene sheets, the corrosion resistance of the ZRE coating could be enhanced more significantly with incorporating even less 3DG. With introducing only 0.1 wt% 3DG, the corrosion current intensity of the resulting 3DG/ZRE coating was reduced to be about 1/10 that of the G/ZRE coating with the same graphene content and 27% of that of the optimized G/ZRE. The corrosion products of the coating were analyzed with the XRD technique. The results indicated that, in contrast to neat ZRE coating, Zn_5_(CO_3_)_2_(OH)_6_ was absent from the corroded 3DG/ZRE coating, confirming its improved long-term anti-corrosion performance. The porous interconnected framework and high crystallinity of 3DG could contribute to not only its facilely mixing with epoxy resin, but also its effective incorporation into the conductive network of zinc micro-flakes, thus enhancing the corrosion resistance of its ZRE coating at a lower content. The innovative technology to improve the anti-corrosion performance of the ZRE coatings via using the 3D graphene fillers should be capable to be extended to other 2D fillers, such as MXenes.

## 1. Introduction

Recently, two-dimensional (2D) multi-functional materials, including graphene, graphitic carbon nitride, and MXene, have aroused extensive research interest all over the world, and a large amount of work on their facile fabrication and applications in many fields [1,2,3,4]. Carbon materials, such as the well-known graphene and amorphous carbon coatings, have been applied for the surface protection of various metals [5,6,7]. Due to their unique characters, including an excellent barrier property, high chemical stability, and lubricating behavior, these carbon materials as coatings exhibit a high anti-corrosive and anti-scratch performance. Another method to exploit fully their functional property in the surface protection of metallic substrate is achieved by incorporating them into polymer composite coatings [8,9,10,11,12,13,14,15]. It is suggested that graphene sheets incorporated into a zinc-rich epoxy (ZRE) composite coating will make the best use of the cathodic protection of zinc powders in the coatings through facilitating the construction of a conductive network between zinc particles. Up to now, many types of carbon materials have been investigated for this aim, including one-dimensional (1D) carbon fibers [16] or nanotubes [17], and 2D graphenes [18,19]. 

Due to their high aspect ratio and 2D structure, graphene sheets are ready to aggregate in the polymer matrix, resulting in a great challenge to prepare their composite with a high performance. To alleviate this, graphene derivatives, including graphene oxide or fluorinated reduced graphene oxide, have been developed and used to prepare the composites [20,21,22,23]. However, the derivatives generally have the deteriorated conductivity due to their decreased crystallinity or even being in an amorphous state. 

Graphene foam has been developed in recent years. The most common method to prepare the foam is the so-called chemical vapor deposition method. With this method, the porous structure of graphene is controlled by the metal catalytic foam templates, and the thickness of the graphene foam strut-wall can be controlled to be within nanometers. Due to their unique porous 3D structures, their polymer composites with various advanced properties [24,25,26], including high wear resistance and electrical or thermal conductivity, have been developed and investigated as multi-functional composites [25,26]. However, little information on its application in anti-corrosive zinc-rich coating is available so far. 

In this work, the influence of the spatial structure of high-crystalline graphene fillers on the corrosion resistance of ZRE coatings was studied. Besides conventional 2D graphene sheets, 3DG with a porous structure and interconnected graphene framework has been applied to modify the ZRE paint. Both the morphology and physical chemical properties of the as-prepared 3DG will be characterized. To avoid the influence of the crystalline structure of graphene on the anti-corrosive behavior of the modified ZRE coating, 3DG with a similar crystalline structure to graphene sheets has been used in this study. The anti-corrosion behaviors of the ZRE modified with different graphenes in the artificial simulated seawater (3.5 wt% NaCl solution) will be studied by the potentiodynamic polarization test and electrochemical impedance spectroscopy, as well as the long-term immersion test.

## 2. Materials and Methods

### 2.1. Materials

Commercial graphene sheets (G, lateral size of 10~50 µm and thickness of 3–8 nm) were purchased from Suzhou Tanfeng Technology Co. Ltd. (Suzhou, China). Epoxy (E-44) was provided by Xiya Reagent Co. (Shangdong, China). Xylene, n-butanol, and polyamide 651 were all chemically pure and purchased from McLean Group. (McLean, VA, USA) Commercial zinc flakes with a lateral size of 5–15 µm were ordered from Xiamen Empire New Material Technology Co. Ltd. (Xiamen, China) and a Q235 steel plates with a size of 30 mm × 40 mm were provided by Fuquan Metal Co. Ltd. (Quanzhou, China).

### 2.2. Preparation of Composite Paint and Coating Samples

**Scheme 1:**
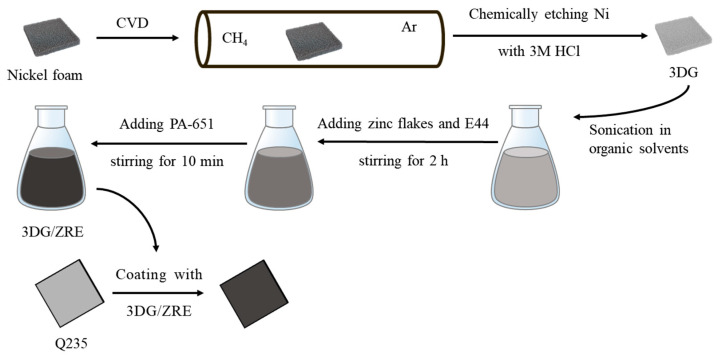
Schematic diagram of the process for the preparation of 3DG/ZRE composite and its coating on the Q235 steel plate.

The whole process was illustrated as Scheme 1. As depicted in previous work [25], graphene foam was produced by the template-directed chemical vapor deposition (CVD) method following the removal of Ni foam. It was then used to prepare 3DG/ZRE paint. The epoxy composite paint comprised two parts, i.e., the component A (epoxy resin and Zn powder) and component B (polyamine curing agent, PA-651). For the fabrication of the modified paint, common graphene or graphene foam was firstly dispersed into epoxy resin diluent ultrasonically. After adding E-44 and Zn powder, the dispersion was magnetically stirred for 2 h. The paint was fabricated finally by mixing it with component B. In the paint composite system, organic solvent (xylene: n-butanol = 7:3) was used as the epoxy diluent and its mass fraction in the paint was 20 wt%. The mass fraction of E-44, zinc powder, and PA-651 is 30 wt%, 35 wt%, and 15 wt%, respectively. Depending on the fraction of graphene (0.1 wt%, 0.3 wt%, 0.5 wt%, and 0.7 wt%) or 3 DG (0.1 wt%), the resulted coatings were labeled as ‘the fraction G/ZRE’ or ‘0.1 wt% 3DG/ZRE’, respectively. Prior to being coated with the ZRE paint, Q235 carbon steel plates were sandblasted and washed with acetone. The paint was applied to the surface of the sandblasted Q235 plates by a rod applicator and cured in an oven at 60 °C for 24 h. Moreover, neat ZRE coating was prepared using the same method in the absence of any graphene filler. 

### 2.3. Characterization Methods

The crystalline structures of the graphene samples were characterized with an X-ray diffraction analyzer (Rigaku Ultima IV, Nagano, Japan). The carbon structure of 3DG was analyzed with a Raman spectrometer (Invia Renishaw Raman, Vignate Gieres, France). The morphology of the carbon sample was observed with a scanning electron microscope (SEM; Hitachi S4800, Tokyo, Japan). An optical microscope (DM3000) was also utilized to observe the morphology of graphene fillers in epoxy resin.

The corrosion measurements of the ZRE coatings were measured on a CHI 760e electrochemical analyzer. A three-electrode half-cell system was constructed with a saturated calomel electrode (SCE) as the reference electrode, platinum plate as the auxiliary electrode, and ZRE coated Q235 plates as the working electrode. First of all, an open circuit potential test (OCP) of the sample was performed to ensure it reached a relative steady state. The potentiodynamic polarization test was carried out at a scan rate of 10 mV s^−1^ and its potential range was set to be ±250 mV around the OCP. Three potentiodynamic polarization tests were carried out for both neat ZRE and 3DG/ZRE samples and the typical ones are reported in the work. The electrochemical impedance spectroscopy (EIS) tests were performed in a frequency range of 10 kHz~0.1 Hz at room temperature. The simulated seawater (3.5 wt% NaCl solution) was used as the corrosive electrolyte. 

## 3. Results and Discussion

### 3.1. Structure Characterization of G and 3DG

**Figure 1:**
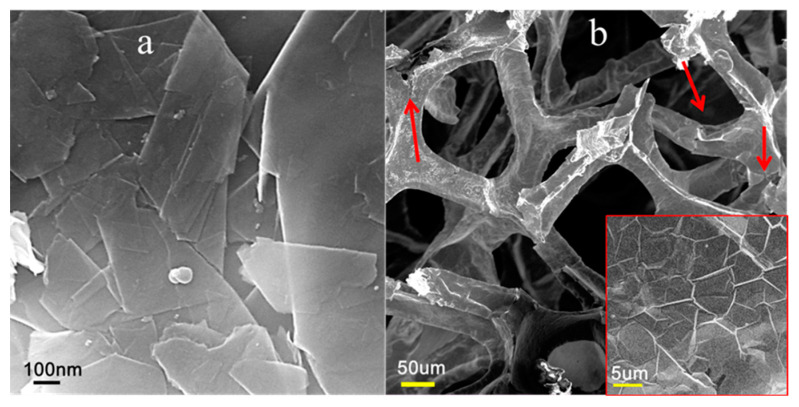
SEM images of graphene sheets (**a**) and 3DG (**b**).

Different from the common 2D morphology of normal graphenes (Figure 1a), 3DG is a porous graphene monolith, which is composed of an interconnected hollow graphene framework (highlighted by red arrows in Figure 1b). Such a rich porous structure results from the metal framework template, which acts as the catalytic substrate for continuously growing graphene. Similar to the 3D porous amorphous carbon fabricated with the flame coating of Ni foam [27], grain boundaries replicating Ni substrate could be clearly observed in the high-magnification SEM image of the 3DG strut surface (see the inset in Figure 1b). Evidently, the graphene surface of 3DG is totally different from the conventional 2D graphene products, whose smooth surface is clearly shown as Figure 1a. These grain boundaries of 3DG may be helpful to improve their interface with epoxy matrix. Due to the porous framework and interconnected hollow strut structure of 3DG and the well-known capillary action, organic liquids could mixed well with it, as such liquids could infiltrate easily into the porous 3DG.

**Figure 2:**
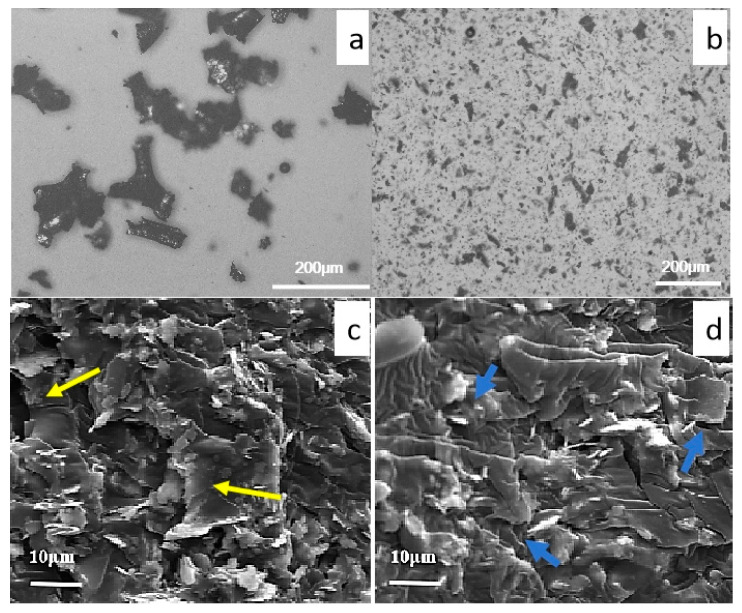
Optical image of the carbon fillers dispersed in epoxy resin: (**a**) 3DG and (**b**) graphene sheets; SEM images of the ZRE coatings: (**c**) 3DG/ZRE and (**d**) G/ZRE.

Under the magnetic stirring in epoxy resin, the graphene framework monolith infiltrated with epoxy resin will be in situ broken into small fragment particles, whose morphology will have 3D irregular characteristics with a size larger than that of the conventional 2D graphene sheets [24]. As shown in Figure 2a, 3DG fragments are dispersed in the epoxy resin after the mixing process and they are much larger than those common graphene sheets in Figure 2b. It is noted that some struts could be observed occasionally in Figure 2a. The SEM image of the 3DG/ZRE composite confirms the presence of the large size of 3DG fragments, which are highlighted by the yellow arrows in Figure 2c. Evidently, they are surrounded by small Zn flakes in the composites. In contrast, it can be seen that small graphene sheets (highlighted by blue arrows in Figure 2d) appear together with Zn flakes, making it hard to define them from each other sometimes in the G/ZRE composites due to their similar 2D morphology. The large size and unique spatial structure of the 3DG fragments are expected to not only alleviate the re-stack of graphene sheets in the polymer matrix, but also favor the formation of the electric conductive path between Zn micro-flakes in the ZRE coatings, which helps to fully exert their cathodic protection function.

**Figure 3:**
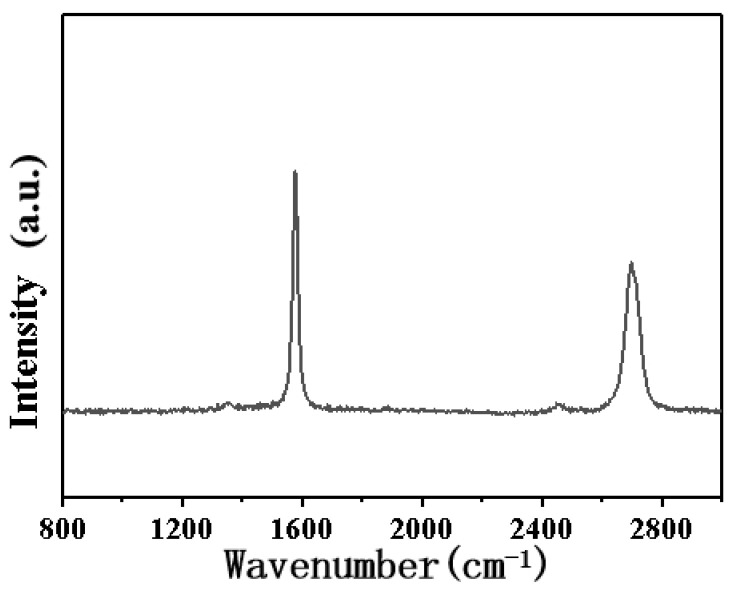
Raman spectrum of 3DG.

Similar to the research on the graphene deposited on catalytic metal substrates in the literature [6,28], two strong peaks for the G band at 1576.7 cm^−1^ and the 2D band at 2700 cm^−1^, together with a very weak peak for the D band around 1357 cm^−1^, could be observed in the Raman spectrum of 3DG (Figure 3). As the D band refers to the disorder carbon structure, and the relative intensities I_D_/I_G_ could be used to evaluate the defect degree of carbonaceous structure, the Raman spectrum of 3DG confirms its high crystalline structure. Moreover, the intensity ratio (I_2D_/I_G_) of 3DG is 0.66, which is much less than 1, implying its multi-layered structure [6,7,29].

**Figure 4:**
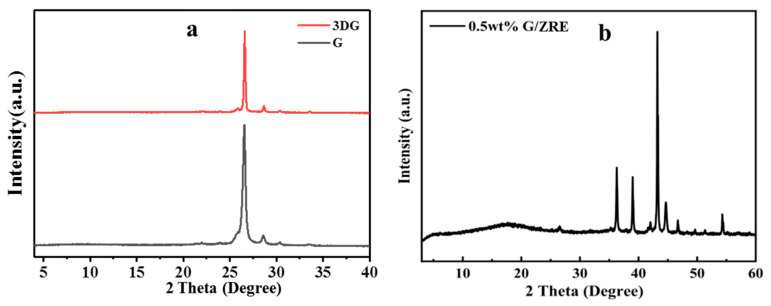
XRD patterns of (**a**) graphene sheets and 3DG, (**b**) 0.5 wt% G/ZRE.

Figure 4a shows the XRD patterns of both 3DG and normal 2D graphene (G). It can be seen that 3DG displays a strong peak around 26°, which belongs to the (002) of graphite. Its pattern is similar to that of common 2D graphene sheets, suggesting that both samples have a high crystalline structure. Moreover, based on the strong peak in XRD patterns in Figure 4a, the average crystallite size of 3DG is calculated to be 40.5 nm, which is much larger than that of graphene sheets (19.8 nm). Furthermore, 3DG displays a much smaller lattice strain (0.000856) and dislocation density (0.000609) than graphene sheets, whose counterpart value is 0.002550 and 0.001752, respectively. These results demonstrate the high-quality continuous graphene struts in 3DG, which favor their electrical linkage with zinc particles in the ZRE coating. In the graphene-modified ZRE coating, quite a few strong peaks appear around 40° (Figure 4b). They majorly originate from the large amount of Zn flakes in the coating. Due to the low content of graphene in the composite, the peak contributed from graphene (26°) is too weak to be defined. For 3DG/ZRE samples, its XRD pattern is almost the same as that of 0.5 wt% G/ZRE.

### 3.2. Corrosion Inhibition of the Coatings

#### 3.2.1. Corrosion Inhibition of Graphene Modified ZRE Coatings

**Figure 5:**
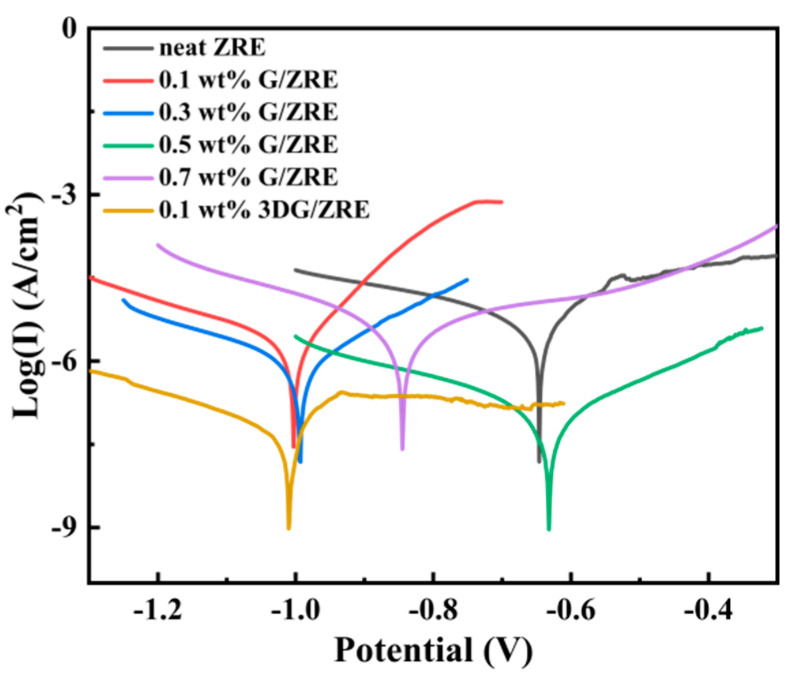
Tafel curves of different ZRE-coated Q235 steel plates in simulated seawater.

The potentiodynamic polarization tests have been performed to characterize the corrosion inhibition behavior of various ZRE coatings. As shown in Figure 5, when incorporating G in the ZRE coating, the Tafel curves of all the samples move downwards, indicating the decreased corrosion current density (Icorr). Evidently, the lowest curve of the G/ZRE coating is the one containing 0.5 wt.% G. Based on Figure 5, the Icorr, corrosion potential (Ecorr), and polarization resistance (Rpo) of the coatings modified with different G content are obtained, and they are listed in Table 1. The Icorr value of neat ZRE is comparable with the other ZRE coating [30]. It can be found that, with the modification by only 0.1 wt.% G, the corrosion inhibition performance of the coating is already enhanced greatly, as its Icorr becomes to be only 1/3 that of neat ZRE coating. With the G content increasing from 0.1 to 0.5 wt.%, the Icorr of its ZRE coating decreases correspondingly (Figure 5). At 0.5 wt.% G, the Icorr of its ZRE coating is the lowest (7.14 × 10^−7^ A cm^−2^), which means a ~10-fold improvement in the corrosion resistance, comparing with that of neat ZRE (6.1 × 10^−6^ A cm^−2^). However, when further increasing the G content to 0.7 wt.%, the Icorr of its ZRE coating increases (Table 1). The increased Icorr of the coating could be due to the aggregation of G at high content, which deteriorates the barrier property of the coating. Additionally, the 0.5 wt.% G/ZRE coating displays the largest Rpo among all the G/ZRE samples. As a result, 0.5 wt.% is determined to be the optimal G content for modifying ZRE coating.

#### 3.2.2. Corrosion Resistance of 3DG Modified ZRE Coatings

For the first time, it was revealed that the anti-corrosion performance of ZRE coating was capable to be further enhanced by replacing common G with 3DG, which exhibits a similar high crystalline structure. Due to its 3D porous structure, only 0.1 wt% 3DG is needed to modify the ZRE coating. As shown in Figure 5, its Tafel test curve moved downwards a little bit, along with the decreased Icorr, relative to that of 0.5 wt% G/ZRE. Its Icorr was 1.9 × 10^−7^ A cm^−2^, which was reduced to be nearly one quarter that of 0.5 wt% G/ZRE, as shown in Table 1. Its Rpo also became much larger than that of 0.5 wt% G/ZRE. These results demonstrate the greatly positive effect of 3DG in promoting the corrosion resistance of the ZRE coating.

**Figure 6:**
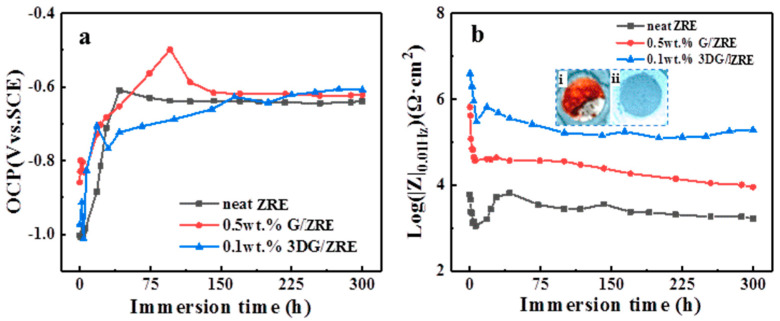
The dependence of (**a**) OCP and (**b**)|Z|_0.01Hz_ on the immersion time for neat ZRE-, G/ZRE-, and 3DG/ZRE-coated Q235 steel plates.

The long-term corrosion resistance behavior of the ZRE coating was evaluated by the immersion test. It was suggested that the open circuit voltage (OCP) of the ZRE coating could be used as a factor to evaluate their protection performance [31]. Figure 6a shows the variation of OCP values of the three ZRE through the 300 h immersion test. Initially, the values of all the coating samples are far lower than −0.6 V. All the OCP values of the ZRE coatings increase quickly within the first 2-day immersion. This could be due to the oxidation of zinc particles and the formation of the corresponding compounds as the corrosive species continuously penetrate into the ZRE coating. Theoretically, the cathodic protection function of the coating will be reduced by the generation of the less conductive corrosion products due to the destruction of electrical linkage between zinc particles. Comparing with neat ZRE and G/ZRE, it takes much more time (~170 h) for 3DG/ZRE to reach a relative stable value, which means more zinc particles are involved in the sacrificial anode-based cathodic protection process. These results confirm the pronounced effect of 3DG fillers on maximizing the cathodic protection of ZRE coating.

**Figure 7:**
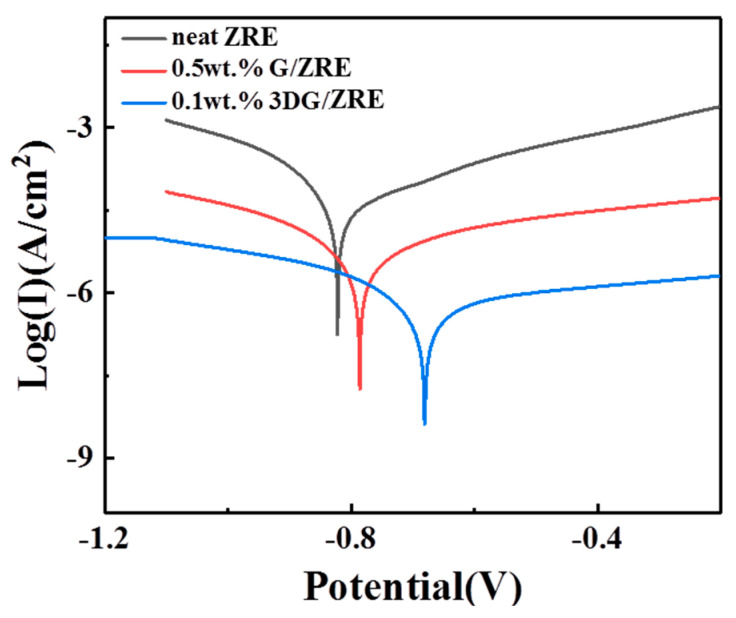
Tafel curve of neat ZRE-, G/ZRE-, and 3DG/ZRE-coated Q235 steel plates after 300 h immersion in simulated seawater.

The dependence of |Z|_0_._01Hz_ of the ZRE coatings on the time is shown in Figure 6b, where the |Z|_0_._01Hz_ of all the coatings decreased quickly with the immersion time initially. Obviously, 0.5 wt% G/ZRE exhibited a larger |Z|_0_._01Hz_ than neat ZRE during the whole immersion test, confirming the positive effect of incorporating G on promoting the corrosion inhibition of ZRE coating. Additionally, the curve of 0.1 wt% 3DG/ZRE is always higher than that of 0.5 wt% G/ZRE all through the immersion process, demonstrating the superior effect of 3DG over the 2D graphene on enhancing the corrosion resistance of ZRE coating. The digital image of the exposed circle area of neat ZRE after the immersion test indicates that its major part is brown, together with some white areas (the inset photo i in Figure 6b). The brown area is caused by the Fe^3+^-containing corrosion product of steel substrate and the white area should be attributed to the zinc-containing corrosion products, which confirm the severe failure of the coating. In contrast, such a phenomenon is totally absent in the 0.1 wt% 3DG/ZRE sample (the inset photo ii in Figure 6b). Furthermore, after 300 h immersion in the simulated seawater, the Tafel curve of the 0.1 wt% 3DG/ZRE exhibits the smallest Icorr among all the three ZRE samples, as shown in Figure 7 and Table 2. Its Icorr (5.2 × 10^−7^ A cm^−2^) is nearly 1/10 that of 0.5 wt% G/ZRE (4.02 × 10^−6^ A cm^−2^) (Table 2). Additionally, its Rpo is remarkably larger than that of 0.5 wt% G/ZRE as well. All of these experimental results confirm the significantly improved corrosion resistance of 0.1 wt% 3DG/ZRE.

**Figure 8:**
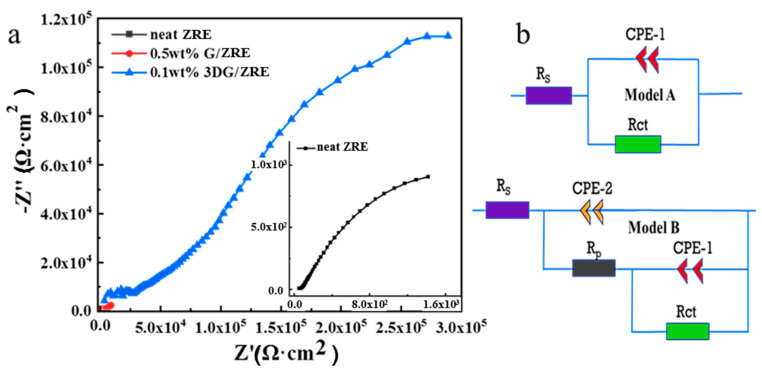
(**a**) Nyquist diagrams of neat ZRE-, G/ZRE-, and 3DG/ZRE-coated Q235 steel plates after 300 h immersion in the simulated seawater and (**b**) the equivalent circuit.

The Nyquist plots of the ZRE coatings after being soaked in simulated seawater are presented in Figure 8a. The equivalent circuit models used for the analysis of EIS data are illustrated in Figure 8b, where Rs represents the electrolyte resistance and Rp and CPE-1 represent the ZRE coating pore resistance and constant phase element of the ZRE capacitor, respectively, whereas Rct and CPE-2 refer to the constant phase element and charge transfer resistance of double-layer capacitor [30,32]. The fitted data for the three ZRE coatings are listed in Table 3. Rct could be used to evaluate a coating’s corrosion resistance and a high Rct value always meant a low-speed corrosion reaction. It can be found that the Rct of 0.1 wt% 3DG/ZRE (5.99 × 10^5^ Ω·cm^−2^) was ~46 times that of 0.5 wt% G/ZRE (1.29 × 10^4^ Ω·cm^−2^), which agreed well with the aforementioned Tafel test results (Table 1). This implies the greatly decelerated corrosion of the ZRE coating with the modification of 3DG. Moreover, the larger Rp value generally meant a better physical barrier property of a coating [30]. The Rp of 0.1 wt% 3DG/ZRE (3.59 × 10^4^ Ω·cm^−2^) was ~10 times that of 0.5 wt% G/ZRE (3.53 × 10^3^ Ω·cm^−2^) (Table 3), indicating the significant effect of 3DG on improving the anti-corrosion property of the ZRE coating. Additionally, the smaller CPE-2 of 0.1 wt% 3DG/ZRE coincided this conclusion, as this capacitance was suggested to correspond to the barrier properties of the coatings at a high frequency [33]. The positive effect of 3DG should be related to its unique 3D structure, which facilitates its mixing with epoxy, hinders the permeation of the corrosives, and interconnects Zn particles, thus enhances the corrosion resistance of its ZRE coating.

**Figure 9:**
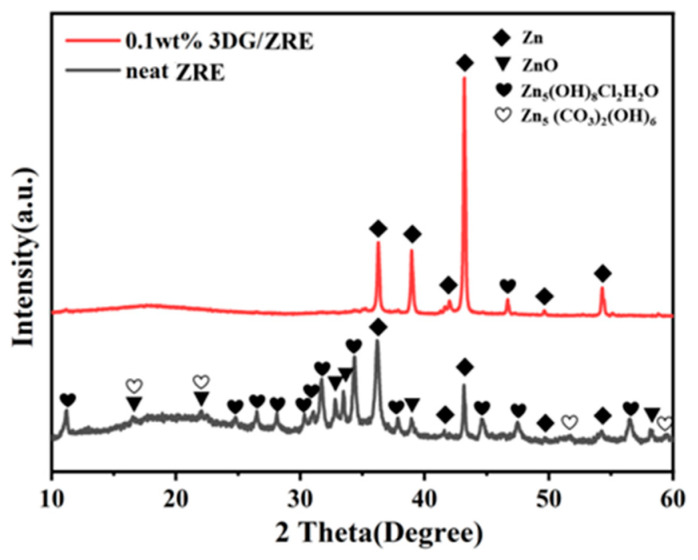
XRD patterns of neat ZRE- and 3DG/ZRE-coated Q235 steel plates after 300 h immersion in simulated seawater.

As suggested in the literature [34,35], the electrochemical reactions related to the zinc-containing products during the corrosion process of ZRE are given as the following Equations (1)–(9). Besides ZnO/Zn(OH)_2_ products (Equations (1)–(5)), the insoluble complexes, including Willemite (Equations (6)) and hydrozincite (Equations (7)–(9)), could be also formed under certain conditions (pH, the concentration of Cl^−^ and dissolved CO_2_). After the continuous 300 h immersion test, the Q235 steel plates coated with either neat ZRE or 3DG/ZRE were taken out from the simulated seawater and their XRD patterns were taken to characterize the corrosion products. Consistent with the zinc corrosion products reported previously [36,37], many peaks in the XRD pattern of the soaked neat ZRE sample (Figure 9) could be attributed to ZnO, Zn_5_(OH)_8_Cl_2_H_2_O, and Zn_5_(CO_3_)_2_(OH)_6_, respectively. Interestingly, different from the presence of both complicated complex products on the soaked neat ZRE coating, no peak contributed from hydrozincite Zn_5_(CO_3_)_2_(OH)_6_ could be found on the pattern of the 3DG/ZRE coating after the 300 h immersion test. This could be due to the excellent barrier property of 3DG particles to carbon dioxide, demonstrating its great contribution to the surface protection of the ZRE coating.

Zn→Zn^2+^ + 2e^−^ (1)

Zn^2+^ + 2OH^−^→Zn(OH)_2_ (2)

Zn(OH)_2_→ZnO + H_2_O (3)

Zn(OH)_2_ + 2OH^−^→Zn(OH)_4_^2−^ (4)

Zn(OH)_4_^2−^→ZnO + H_2_O + 2OH^−^ (5)

5Zn(OH)_2_ + H_2_O + 2Cl^−^→Zn_5_(OH)_8_Cl_2_·H_2_O + 2OH^−^ (6)

5Zn(OH)_2_ + 2CO_2_→Zn_5_(CO_3_)_2_(OH)_6_ + 2H_2_O (7)

5ZnO + 2CO_2_ + 3H_2_O→Zn_5_(CO_3_)_2_(OH)_6_ (8)

5Zn(OH)_4_^2−^ + 2CO_2_→Zn_5_(CO_3_)_2_(OH)_6_ + 2H_2_O + 10OH^−^ (9)

**Figure 10:**
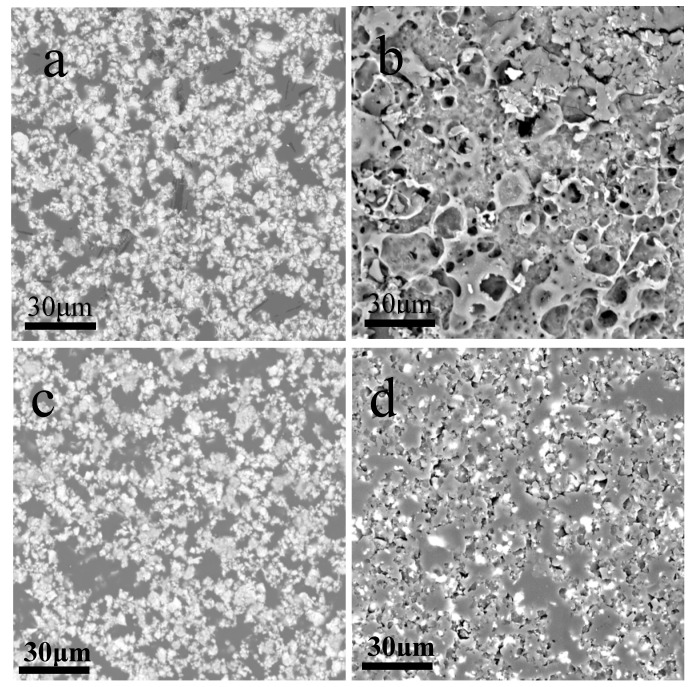
SEM images of ZRE-coated Q235 steel samples in simulated seawater: neat ZRE before (**a**) and after (**d**) 300 h immersion; 3DG/ZRE before (**c**) and after (**d**) 300 h immersion.

As shown in Figure 10d, after the immersion test, the corroded surface of the 3DG/ZRE coating became a little rougher than that before the test (Figure 10c) and small pores could be observed. These could be due to the oxidation of zinc particles in the coating and the formation of ZnO and Zn_5_(OH)_8_Cl_2_H_2_O, as mentioned above. In contrast, a more significant morphology change occurred for the neat ZRE-coated sample after the immersion test, as indicated in Figure 10a,b. Both the larger pores and the crack in Figure 10b confirm the seriously corroded neat ZRE-coated Q235 steel plate, which are inconsistent with the digital images shown in Figure 6 and the XRD analysis results mentioned above. 

## 4. Conclusions

In this work, conventional 2D graphene sheets and 3DG fillers were used to modify the ZRE composite to improve the corrosion resistance of the ZRE coatings. These main conclusions are drawn:

(1) The corrosion resistance of the ZRE coating could be improved by incorporating common 2D graphene, and the optimal G content is 0.5 wt%.
(2) The corrosion resistance of the ZRE coating could be further enhanced significantly by incorporating only 0.1 wt% 3DG.
(3) Long-term immersion tests confirmed the superior effect of 3DG on improving the surface protection performance of ZRE as well.

This work revealed, preliminarily, the significant effect of 3D graphene fillers on the surface protection performance of ZRE coating. However, to understand the spatial structure of graphene fillers on the properties of their ZRE coatings more comprehensively, further work needs to be done, such as the mathematical simulation of the contribution of 3DG to the cathodic protection process of ZRE coating.

## Figures and Tables

**Table 1:** Icorr, Ecorr, and Rpo of different ZRE-coated Q235 steel plates in simulated seawater.

ZRE Samples	Icorr (A·cm^−2^)	Ecorr (V)	Rpo (Ω·cm^2^)
neat ZRE	6.11 × 10^−6^	−0.646	5.67 × 10^3^
0.1 wt% G/ZRE	1.95 × 10^−6^	−1.002	1.31 × 10^4^
0.3 wt% G/ZRE	1.15 × 10^−6^	−0.993	3.21 × 10^4^
0.5 wt% G/ZRE	7.14 × 10^−7^	−0.631	3.65 × 10^5^
0.7 wt% G/ZRE	3.36 × 10^−6^	−0.844	1.27 × 10^4^
0.1 wt% 3DG/ZRE	1.90 × 10^−7^	−1.010	5.04 × 10^5^

**Table 2:** The Icorr, Ecorr, and Rpo of neat ZRE-, G/ZRE-, and 3DG/ZRE-coated Q235 steel plates after 300 h immersion in simulated seawater.

Samples Soaking for 300 h	Icorr (A·cm^−2^)	Ecorr (V)	Rpo (Ω·cm^2^)
neat ZRE	4.87 × 10^−5^	−0.824	7.76 × 10^2^
0.5 wt% G/ZRE	4.02 × 10^−6^	−0.786	9.89 × 10^3^
0.1 wt% 3DG/ZRE	5.20 × 10^−7^	−0.681	8.98 × 10^4^

**Table 3:** Eletrochemical parameters obtained from EIS data of different ZRE-coated Q235 steel.

ZRE Samples	CPE-1 (S·cm^−2^·s^n^)	Rp(Ω·cm^2^)	CPE-2 (S·cm^−2^·s^n^)	Rct (Ω·cm^2^)	Model
neat ZRE	6.84 × 10^−4^	-	-	8.37 × 10^3^	A
0.5 wt% G/ZRE	7.19 × 10^−7^	3.53 × 10^3^	1.03 × 10^−4^	1.29 × 10^4^	B
0.1 wt% 3DG/ZRE	5.02 × 10^−8^	3.59 × 10^4^	1.23 × 10^−5^	5.99 × 10^5^	B

## Data Availability

Experimental data from this study are available upon request.
